# Prevalence of HIV-1 drug resistance in Eastern European and Central Asian countries

**DOI:** 10.1371/journal.pone.0257731

**Published:** 2022-01-21

**Authors:** Alina Kirichenko, Dmitry Kireev, Alexey Lopatukhin, Anastasia Murzakova, Ilya Lapovok, Daria Saleeva, Natalya Ladnaya, Agigat Gadirova, Sabina Ibrahimova, Aygun Safarova, Trdat Grigoryan, Arshak Petrosyan, Tatevik Sarhatyan, Elena Gasich, Anastasia Bunas, Iryna Glinskaya, Pavel Yurovsky, Rustam Nurov, Alijon Soliev, Laylo Ismatova, Erkin Musabaev, Evgeniya Kazakova, Visola Rakhimova, Vadim Pokrovsky

**Affiliations:** 1 Central Research Institute of Epidemiology, Moscow, Russian Federation; 2 Research Institute of Lung Diseases, Baku, Azerbaijan; 3 Republic Center of the Struggle against AIDS, Baku, Azerbaijan; 4 National Center of AIDS Prevention, Yerevan, Armenia; 5 Republican Research and Practical Center for Epidemiology and Microbiology, Minsk, Belarus; 6 Republican Center for Hygiene, Epidemiology and Public Health, Minsk, Belarus; 7 Republican AIDS prevention center, Dushanbe, Tajikistan; 8 Research Institute of Virology, Tashkent, Uzbekistan; 9 Center for development of profession qualification of medical workers, Tashkent, Uzbekistan; University of Cincinnati College of Medicine, UNITED STATES

## Abstract

**Background:**

Eastern Europe and Central Asia (EECA) is one of the regions where the HIV epidemic continues to grow at a concerning rate. Antiretroviral therapy (ART) coverage in EECA countries has significantly increased during the last decade, which can lead to an increase in the risk of emergence, transmission, and spread of HIV variants with drug resistance (DR) that cannot be controlled. Because HIV genotyping cannot be performed in these countries, data about HIV DR are limited or unavailable.

**Objectives:**

To monitor circulating HIV-1 genetic variants, assess the prevalence of HIV DR among patients starting antiretroviral therapy, and reveal potential transmission clusters among patients in six EECA countries: Armenia, Azerbaijan, Belarus, Russia, Tajikistan, and Uzbekistan.

**Materials and methods:**

We analyzed 1071 HIV-1 pol-gene fragment sequences (2253–3369 bp) from patients who were initiating or reinitiating first-line ART in six EECA counties, i.e., Armenia (n = 120), Azerbaijan (n = 96), Belarus (n = 158), Russia (n = 465), Tajikistan (n = 54), and Uzbekistan (n = 178), between 2017 and 2019. HIV Pretreatment DR (PDR) and drug resistance mutation (DRM) prevalence was estimated using the Stanford HIV Resistance Database. The PDR level was interpreted according to the WHO standard PDR survey protocols. HIV-1 subtypes were determined using the Stanford HIV Resistance Database and subsequently confirmed by phylogenetic analysis. Transmission clusters were determined using Cluster Picker.

**Results:**

Analyses of HIV subtypes showed that EECA, in general, has the same HIV genetic variants of sub-subtype A6, CRF63_02A1, and subtype B, with different frequencies and representation for each country.

The prevalence of PDR to any drug class was 2.8% in Uzbekistan, 4.2% in Azerbaijan, 4.5% in Russia, 9.2% in Armenia, 13.9% in Belarus, and 16.7% in Tajikistan. PDR to protease inhibitors (PIs) was not detected in any country. PDR to nucleoside reverse-transcriptase inhibitors (NRTIs) was not detected among patients in Azerbaijan, and was relatively low in other countries, with the highest prevalence in Tajikistan (5.6%). The prevalence of PDR to nonnucleoside reverse-transcriptase inhibitors (NNRTIs) was the lowest in Uzbekistan (2.8%) and reached 11.1% and 11.4% in Tajikistan and Belarus, respectively.

Genetic transmission network analyses identified 226/1071 (21.1%) linked individuals, forming 93 transmission clusters mainly containing two or three sequences. We found that the time since HIV diagnosis in clustered patients was significantly shorter than that in unclustered patients (1.26 years vs 2.74 years). Additionally, the K103N/S mutation was mainly observed in clustered sequences (6.2% vs 2.8%).

**Conclusions:**

Our study demonstrated different PDR prevalence rates and DR dynamics in six EECA countries, with worrying levels of PDR in Tajikistan and Belarus, where prevalence exceeded the 10% threshold recommended by the WHO for immediate public health action.

Because DR testing for clinical purposes is not common in EECA, it is currently extremely important to conduct surveillance of HIV DR in EECA due to the increased ART coverage in this region.

## Introduction

By the end of 2019, the UNAIDS reported that 1.7 million people were living with HIV (PLWH) in the Eastern Europe and Central Asia (EECA) region. This is one of only three worldwide regions where the HIV epidemic continues to grow at a concerning rate, with the number of new HIV infections rising by 72% between 2010 and 2019 [1].

In EECA countries, the management of HIV is associated with major challenges that include a high prevalence of HIV, poor adherence to antiretroviral therapy (ART), stock-outs of ART drugs, the use of nonnucleoside reverse-transcriptase inhibitor (NNRTI)-based first-line ART regimens with a low genetic barrier, and weak routine viral load monitoring systems [1,2].

Numerous studies have demonstrated that ART prevents morbidity and mortality for PLWH and has clear HIV prevention benefits, reducing the number of new infections [3–7].

The majority of countries have adopted the UNAIDS ’90-90-90’ strategy to achieve the ambitious goal of ending the AIDS epidemic, as a public health threat, by 2030, which is related to the expansion of ART [7].

In recent years, EECA countries have adopted the ’90-90-90’ strategy and have officially adopted a “treat-all” and “treat-early” policy and significantly increased ART coverage of patients. Nonetheless, many countries in the region are far from reaching the ’90-90-90’ goal; as of 2019, only 63% of PLWH aware of their status in the region were receiving treatment, leaving only 41% of all PLWH virally suppressed [1].

Unfortunately, the expansion of ART coverage and the use of NNRTI-based first-line ART regimens without the availability of HIV genotyping and viral load tests have inevitably increased the risk of emergence, transmission, and spread of HIV variants with drug resistance (DR) [8–10].

Modeling studies have shown that inaction in response to increasing HIV DR will lead to increases in mortality, HIV incidence, and overall costs of ART regimens, thereby impeding progress towards achieving the end of the epidemic [8–14].

Since 2014, the WHO has recommended that scaling up ART should be accompanied by surveillance of both acquired HIV DR (ADR) in ART-experienced patients and pretreatment HIV DR (PDR) in patients initiating or reinitiating ART [15–17].

Whereas in high-income countries, DR testing is part of routine care for patients starting first-line therapy, in most EECA countries, DR testing is rarely performed only for patients with virologic failure due to the unavailability of the procedure and high costs [2].

In this region, nationwide estimates of DR are not available, and data about the prevalence, pattern, and trend of HIV DR are very sparse and scattered. A number of studies conducted in Russia showed an increase in the prevalence of HIV DR among naïve patients from 0–1.5% in 1998–2014 [18,19] to 5.3% in a recent study [20]. In other EECA countries, there were limited or nonexistent data. Uzbekistan reported a DR prevalence of 2.96% for samples collected in 2015–2016 [21]. In Armenia, the prevalence of HIV DR for samples collected in 2009–2010 was 1.5% [22]. There is no available data about HIV DR in Azerbaijan, Belarus, and Tajikistan.

Therefore, surveillance of the HIV epidemic and HIV DR is strongly needed in this region since increased ART coverage and the subsequent increase in HIV DR to used drugs can significantly worsen the effectiveness of therapy and make it impossible to achieve the stated goals.

It is thus essential that actions to monitor, prevent, and respond to HIV DR in EECA countries are implemented at the laboratory, clinical, and policy levels Considering this need, we conducted the international multicenter, cross-sectional, retrospective, surveillance study REZEDA-1 in six EECA countries: Armenia, Azerbaijan, Belarus, Russia, Tajikistan, and Uzbekistan. The aims of this study were to monitor the prevalence of circulating HIV-1 genetic variants, assess the prevalence of HIV DR among patients starting antiretroviral therapy, and reveal potential transmission clusters.

## Materials and methods

### Study population

Enrollment took place at AIDS clinics in six countries, i.e., Armenia (n = 120), Azerbaijan (n = 96), Belarus (n = 158), Russia (n = 465), Tajikistan (n = 54), and Uzbekistan (n = 178), as part of the cross-sectional, retrospective, surveillance study REZEDA-1 in EECA between January 2017 and February 2019. Study REZEDA-1 was approved by the local ethics committees of the Central Research Institute of Epidemiology (Moscow, Russian Federation), Republic Center of the Struggle against AIDS (Baku, Azerbaijan), National Center of AIDS Prevention (Yerevan, Armenia), Republican Research and Practical Center for Epidemiology and Microbiology (Minsk, Belarus), Republican AIDS prevention center (Dushanbe, Tajikistan), and Research Institute of Virology (Tashkent, Uzbekistan).The informed written consent of each patient who participated in the study was obtained, prior to the sampling and collection of clinical, demographic, and epidemiological data.

To be included, patients had to be at least 18 years old, ART-naïve or have prior drug exposure for treating HIV infection (with interruption for less than three months) or for prophylaxis, and be initiating or reinitiating first-line ART.

Demographic, clinical, and epidemiological data for participants were obtained from the participants’ medical records.

### Country epidemiological and ART profiles

Data were collected from official sources and national guidelines on HIV prevention, diagnosis, treatment, and care for patients [1,23–29].

### RNA extraction and HIV-1 sequencing

An AmpliSens® HIV-Resist-Seq kit (Central Research Institute of Epidemiology, Russia) was used for RNA extraction from blood plasma samples, and for amplification and sequencing of the HIV pol-gene region encoding a protease and part of a reverse transcriptase (2253–3369 bp according HXB-2 strain, GenBank accession number K03455).

Quality assurance of HIV-1 sequences was carried out using the WHO HIV DR quality control tool (http://pssm.cfenet.ubc.ca/who_qc/) before data analysis.

### HIV-1 drug resistance interpretation

The Stanford HIV Resistance Database (HIVdb Program v 8.9–1 and Calibrated Population Resistance Tool) was used to describe and interpret the HIV DR.

DR level was classified according to the Stanford Penalty Score as high (60), intermediate (30–59), or low (15–29).

As recommended by the WHO standard PDR survey protocols [17], sequences with PDR were defined as those with a score of 15 or higher to:

- nucleoside reverse-transcriptase inhibitor (NNRTI): efavirenz (EFV), nevirapine (NVP);
- any nucleoside reverse-transcriptase inhibitor (NRTI): abacavir (ABC), zidovudine (AZT), stavudine (d4T), didanosine (ddI), emtricitabine (FTC), lamivudine (3TC), tenofovir disoproxil fumarate (TDF);
- any protease inhibitor (PI): atazanavir (ATV), darunavir (DRV), lopinavir (LPV).

In this case, we included all mutations considered by the Stanford Resistance Database to estimate the prevalence of drug resistance mutations (DRMs).

### HIV-1 subtyping

HIV-1 subtypes were determined using the Stanford HIV Resistance Database and subsequently confirmed using the HIV BLAST tool (https://www.hiv.lanl.gov/content/sequence/BASIC_BLAST/basic_blast.html).

### Molecular transmission cluster analysis

Phylogenetic analyses were performed with MEGA 6.0 software using the maximum likelihood method with bootstrap (100 replications) and the generalized time reversible model of nucleotide substitution.

Transmission clusters were determined by using Cluster Picker 1.2.1 [30] with a maximum genetic distance threshold of 0.045 nucleotide substitutions per site within the bootstrap support of more than 90%.

### Statistical analysis

Estimates of the prevalence of PDR were calculated with 95% confidence intervals (CIs). Comparisons between groups were performed using χ^2^ tests. To determine the significance of the difference between the means of two unrelated groups, independent t-test was used. All analyses were performed using STATA (v15).

### GenBank accession numbers

The HIV-1 sequences generated from this study are available in the NCBI database with the GenBank accession numbers MH062016- MH062024, MH062026- MH062029, MH062125- MH062138, MK931539- MK931547, MK931784, MK931789- MK931801, MK931912- MK931931, MK931933, MK931934, MK931937, MK931939, MK931940, MK931942, MK931943, MK931945, MK931947- MK931964, MK931996, MK931997, MK931999- MK932016, and MW483846- MW484805.

## Results

### Country epidemiological and ART profiles

In 2019, the estimated percentage of PLWH in the majority of studied countries was low (approximately 0.1%) [1] and reached 0.7% in Russia (Table 1) [23]. The number of people who know their HIV status ranged from 61% in Tajikistan to 80% in Uzbekistan [1]. A large gap between HIV testing and treatment existed in all countries; among people who knew their HIV status, from 50% in Russia and Tajikistan to 63% in Armenia and Belarus were accessing treatment [1,23]. As a result, the number of PLWH who were virally suppressed was extremely low and ranged from 37% and 38% in Tajikistan and Russia, respectively, to 55% in Armenia [1,23].

**Table 1:** HIV testing and treatment in the studied countries in 2019.

Country	Population, million, n	PLWH, n (%)	PLWH who know their status, n (%)	PLWH on treatment, n (%)	PLWH who are virally suppressed, n (%)
Armenia	2.96	3,500 (0.1)	2,700 (77)	2,190 (63)	1,925 (55)
Azerbaijan	10.07	9,700 (0.1)	6,800 (70)	5,086 (52)	4,074 (42)
Belarus	9.41	28,000 (0.3)	22,084 (79)	17,739 (63)	13,440 (48)
Russia	146.75	1,068,839 (0.7)	776,868 (73)	534,990 (50)	408,088 (38)
Tajikistan	9.54	14,000 (0.1)	8,600 (61)	7,055 (50)	5,180 (37)
Uzbekistan	34.04	50,000 (0.1)	39,842 (80)	28,265 (57)	20,179 (40)

According to the guidelines of the countries, ART has been provided to all adults living with HIV regardless of clinical stage and CD4 cell count since 2016 in Russia [24]; since 2017 in Armenia [25]; since 2018 in Azerbaijan [26], Belarus [27], and Uzbekistan [28]; and since 2019 in Tajikistan [29]. To date, all national ART protocols have been brought in accordance with the 2016 WHO recommendations [31].

PI-based ART regimens are used as second-line regimens only [24–29]. In all countries except Russia, EFV-based first-line ART regimens are still used (Table 2). Dolutegravir (DTG) has been commenced in Armenia and Russia as part of the preferred first-line ART regimen. The licensing agreement on generic DTG has included Armenia since 2016 and Belarus and Azerbaijan since 2020. It should be noted that there was only one preferred first-line ART regimen in Azerbaijan.

**Table 2:** ART profiles in the studied countries in 2019.

Country	First-line preferred ART regimen
Armenia	TDF+3TC/FTC+EFV/DTG
Azerbaijan	TDF+FTC+EFV
Belarus	TDF+3TC/FTC+EFV
Russia	ABC/TDF+3TC/FTC+DTG
Tajikistan	TDF+FTC+EFV; AZT+3TC+EFV
Uzbekistan	TDF+3TC/FTC+EFV/NVP;ABC/AZT+3TC+EFV/NVP

### Characteristics of the study cohorts

Demographic, clinical and epidemiological data for participants from the six countries are summarized in S1 Table. The participants’ median ages were similar and ranged from 33.5 in Tajikistan to 38 in Azerbaijan. Male patients prevailed in all countries, with the exception of Uzbekistan, where the sex distribution was equal. The dominant route of HIV-1 transmission in all countries was sexual contact, mostly heterosexual, followed by intravenous drug use (IDU). The highest IDU transmission rate was found in Belarus (29.7%).

The majority of patients had no ART experience (1037/1071; 96.8%). The majority of patients had their first positive immunoblot between 2015 and 2018.

### HIV-1 subtype prevalence

Sub-subtype A6 (formerly FSU-A or IDU-A) was dominant in Armenia, Azerbaijan, Belarus, and Russia, being found in 79.2%, 88.5%, 93.7%, and 77.8% of patients, respectively [32,33].

Another main genetic variant was the circulating recombinant form (CRF) 63_02A1, which was found in Tajikistan (59.2%) and Uzbekistan (49.4%) and dominated in Tajikistan.

Follow most represented was subtype B in the major countries; its prevalence was 10% in Armenia, 5.2% in Azerbaijan, 3.8% in Belarus, 9.5% in Russia, and 5.6% in Tajikistan.

The genetic variant CRF63_02A1, which apparently originated from Uzbekistan [34] and further spread to EECA countries, was detected in all countries except Belarus. Subtype G was identified only in Russia. Subtype C, which is responsible for the majority of infections worldwide [35], was found only in several patients in Armenia and Uzbekistan.

The highest genetic diversity of HIV-1 was observed in Russia and Armenia. The subtype distribution is summarized in Table 3.

**Table 3:** The distribution of HIV-1 subtypes in six EECA countries.

Subtype, n (%)	Country					
	Armenia	Azerbaijan	Belarus	Russia	Tajikistan	Uzbekistan
**A1**	0	0	1 (0.6)	1 (0.2)	0	0
**A6**	**95 (79.2)**	**85 (88.5)**	**148 (93.7)**	**362 (77.8)**	**18 (33.3)**	**86 (48.3)**
**B**	12 (10)	5 (5.2)	6 (3.8)	44 (9.5)	3 (5.6)	1 (0.6)
**C**	2 (1.7)	0	0	0	0	2 (1.1)
**G**	0	0	0	11 (2.4)	0	0
**CRF02_AG**	0	0	0	1 (0.2)	0	1 (0.6)
**CRF03_AB**	2 (1.7)	0	3 (1.9)	8 (1.7)	1 (1.9)	0
**CRF20_BG**	0	0	0	1 (0.2)	0	0
**CRF63_02A1**	8 (6.6)	6 (6.3)	0	36 (7.8)	**32 (59.2)**	**88 (49.4)**
**CRF06_cpx**	1 (0.8)	0	0	1 (0.2)	0	0

* The numbers in boldface are for the main genetic variant in each country.

### Prevalence of PDR

**Fig 1:**
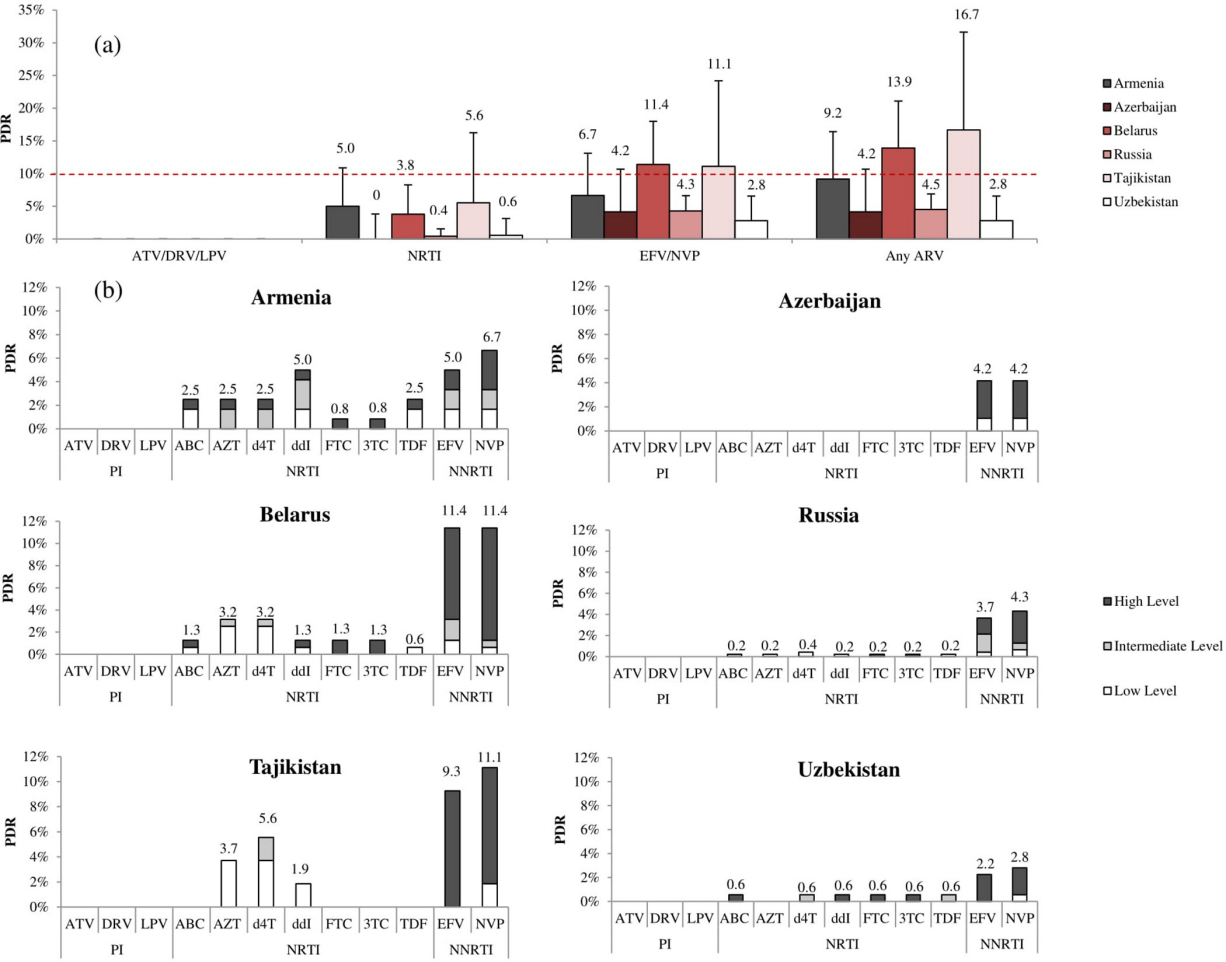
The prevalence of PDR in the six countries according to (a) drug class and (b) antiretroviral drug. The lines represent the 95% CI. ATV, atazanavir; DRV, darunavir; LPV, lopinavir; ABC, abacavir; AZT, zidovudine; d4T, stavudine; ddI, didanosine; FTC, emtricitabine; 3TC, lamivudine; TDF, tenofovir disoproxil fumarate; EFV, efavirenz; NVP, nevirapine. The WHO recommended 10% threshold is indicated by the red dotted line.

According to WHO definitions, the prevalence of PDR to any drug class was 2.8% (95% CI, 0.9%-6.6%) in Uzbekistan, 4.2% (95% CI, 1.1%-10.7%) in Azerbaijan, 4.5% (95% CI, 2.8% -6.9%) in Russia, 9.2% (95% CI, 4.6%-16.4%) in Armenia, 13.9% (95% CI, 8.7%-21.1%) in Belarus, and 16.7% (95% CI, 7.6%-31.6%) in Tajikistan (Fig 1A).

PDR to PIs (LPV, ATV, DRV) was not detected in any country.

PDR to NRTIs was not detected in patients in Azerbaijan, and was relatively low in other countries: 0.4% (95% CI, 0.1%-1.6%) in Russia, 0.6% (95% CI, 0%-3.1%) in Uzbekistan, 3.8% (95% CI, 1.4%-8.3%) in Belarus, 5% (95% CI, 1.8%-10.9%) in Armenia, and 5.6% (95% CI, 1.1%-16.2%) in Tajikistan.

More importantly, the prevalence of PDR to NNRTIs (EFV, NVP) was 2.8% (95% CI, 0.9%-6.6%) in Uzbekistan, 4.2% (95% CI, 1.1%-10.7%) in Azerbaijan, 4.3% (95% CI, 2.6%-6.6%) in Russia, and 6.7% (95% CI, 2.9%-13.1%) in Armenia and reached 11.1% (95% CI, 4.1%-24.2%) and 11.4% (95% CI, 6.8%-18%) in Tajikistan and Belarus, respectively.

Significant differences were observed in the prevalence of PDR to single drugs between countries (Fig 1B). In Armenia, PDR was to all NRTIs with higher to ddI (5.0%; 95% CI, 1.8%-10.9%), and the prevalence of PDR to EFV and NVP was 5.0% (95% CI, 1.8%-10.9%) and 6.7% (95% CI, 2.9%-13.1%), respectively. In Azerbaijan, only the prevalence of PDR to EFV and NVP was determined (4.2%; 95% CI, 1.1%-10.7%). In Belarus, PDR was defined to all NRTIs with more frequently to AZT and d4T (3.2%; 95% CI, 1%-7.4%); the prevalence of PDR to EFV and NVP was 11.4% (95% CI, 6.8%-18%), and the level of DR was predominantly high. In Russia, PDR to all NRTIs was found, with the prevalence not exceeding 0.5%; the prevalence of PDR to EFV and NVP was 3.7% (95% CI 2.1%-5.9%) and 4.3% (95% CI 2.6%-6.6%), respectively.

In Tajikistan, NRTI PDR was determined to ddI (1.9%; 95% CI 0%-10.3%), AZT (3.7%; 95% CI 0.4%-13.4%) and d4T (5.6%; 95% CI 1.1%-16.2%); the prevalence of PDR to EFV and NVP was 9.3% (95% CI, 3%-21.6%) and 11.1% (95% CI, 4.1%-24.2%), respectively, and the level of DR was mostly high. In Uzbekistan, the prevalence of PDR to NRTIs was determined for all drugs (0.6%; 95% CI, 0%-3.1%) except AZT; the prevalence of PDR to the NNRTIs EFV and NVP was low, at 2.2% (95% CI, 0.6%-5.8%) and 2.8% (95% CI, 0.9%-6.6%), respectively.

The prevalence of PDR to only first-line ART-preferred regimen drugs is presented in S2 Table.

As expected, the overall PDR prevalence was higher in ART initiators with prior ART drug exposure than in patients with no previous exposure (6/34, 17.6% vs 66/1037, 6.4%; p = 0.022).

Prevalence of DRMs.

The most prevalent PI DRMs were M46I (3/120; 2.5%) and L10F (2/120; 1.7%) in Armenia and N88D (2/96; 2.1%) in Azerbaijan. In other countries, the prevalence of PI DRMs was determined to be low (<1%).

A62V is a polymorphic mutation for sub-subtype A6 and was the most frequent NRTI DRM in Russia (174/465; 37.4%), Armenia (34/120; 28.3%), Azerbaijan (24/96; 25.0%), Belarus (21/158; 13.3%), and Uzbekistan (48/178; 27.0%). In Tajikistan, the prevalence of A62V was found to be low (2/54; 3.7%). M41L in addition to A62V was found in Belarus (3/158; 1.9%) and Tajikistan (2/54; 3.7%). T69D and L210W were also found in Armenia (3/120; 2.5%).

The most frequent NNRTI DRM in Russia (32/465; 6.9%), Azerbaijan (7/96; 7.3%), and Uzbekistan (7/178; 3.9%) was E138A; this mutation was also found in Armenia (5/120; 4.2%) and Belarus (9/158; 5.7%); in Tajikistan, another substitution, i.e., E138G was found (1/54; 1.9%). In Armenia, the most prevalent NNRTI DRM was V106I (7/120; 5.8%), which was also found in Russia (7/465; 1.5%), Azerbaijan (2/96; 2.1%), and Tajikistan (1/54; 1.9%). K103N was the most common DRM in Belarus (11/158; 7%) and Tajikistan (5/54; 9.3%), and was also observed in Russia (7/465; 1.5%), Azerbaijan (3/96; 3.1%), and Uzbekistan (3/178; 1.7%).

The full list of DRMs is presented in Table 4. The prevalence of NNRTI drug resistance mutations is shown in S2 Fig.

**Table 4:** Prevalence of drug resistance mutations.

Class of ART drugs	DRMs	Country of origin					
		Armenia	Azerbaijan	Belarus	Russia	Tajikistan	Uzbekistan
**PI**	*L10F*	2 (1.7%)	0	0	1 (0.2%)	0	0
	*K20T*	0	0	0	3 (0.6%)	0	0
	*L33F*	0	0	0	3 (0.6%)	0	0
	*K43T*	0	0	0	3 (0.6%)	0	1 (0.6%)
	**M46IL**	3 (2.5%)	1 (1.0%)	0	1 (0.2%)	0	1 (0.6%)
	*Q58E*	0	0	1 (0.6%)	1 (0.2%)	0	1 (0.6%)
	**G73S**	0	0	0	0	0	1 (0.6%)
	*T74P*	1 (0.8%)	0	0	0	0	0
	**N83D**	0	1 (1.0%)	0	0	0	0
	**I85V**	0	0	0	2 (0.4%)	0	0
	**N88D**	0	2 (2.1%)	0	0	0	0
	*L89V*	0	0	0	0	0	1 (0.6%)
**NRTI**	*E40F*	1 (0.8%)	0	0	0	0	0
	**M41L**	1 (0.8%)	0	3 (1.9%)	1 (0.2%)	2 (3.7%)	0
	*E44D*	0	1 (1.0%)	1 (0.6%)	1 (0.2%)	0	2 (1.1%)
	*A62V*	34 (28.3%)	24 (25.0%)	21 (13.3%)	174 (37.4%)	2 (3.7%)	48 (27.0%)
	**K65R**	0	0	0	0	0	1 (0.6%)
	**D67AN**	1 (0.8%)	0	2 (1.3%)	1 (0.2%)	0	0
	**T69D**	3 (2.5%)	0	1 (0.6%)	0	0	0
	**K70QR**	1 (0.8%)	0	1 (0.6%)	1 (0.2%)	0	0
	**V75AM**	1 (0.8%)	0	0	0	1 (1.9%)	0
	**F77L**	0	0	0	0	1 (1.9%)	0
	**M184VI**	1 (0.8%)	0	2 (1.3%)	1 (0.2%)	0	1 (0.6%)
	**L210W**	3 (2.5%)	0	0	0	0	0
	**T215DY**	3 (2.5%)	0	0	0	0	0
	**K219QE**	1 (0.8%)	0	1 (0.6%)	0	0	0
**NNRTI**	*A98G*	2 (1.7%)	0	1 (0.6%)	2 (0.4%)	0	0
	**K101EP**	0	0	1 (0.6%)	0	0	2 (1.1%)
	**K103NS**	2 (1.7%)	3 (3.1%)	11 (7.0%)	14 (3.0%)	5 (9.3%)	3 (1.7%)
	*V106I*	7 (5.8%)	2 (2.1%)	2 (1.3%)	7 (1.5%)	1 (1.9%)	2 (1.1%)
	*V108I*	3 (2.5%)	0	0	2 (0.4%)	1 (1.9%)	1 (0.6%)
	*E138A*	5 (4.2%)	7 (7.3%)	9 (5.7%)	32 (6.9%)	0	7 (3.9%)
	*E138GKQ*	0	1 (1.0%)	2 (1.3%)	1 (0.2%)	1 (1.9%)	1 (0.6%)
	**V179DELT**	2 (1.7%)	3 (3.1%)	5 (3.2%)	7 (1.5%)	0	3 (1.7%)
	**Y181CV**	2 (1.7%)	0	2 (1.3%)	0	0	0
	**G190AS**	1 (0.8%)	0	3 (1.9%)	0	0	1 (0.6%)
	**P225H**	0	0	1 (0.6%)	0	0	1 (0.6%)
	*K238T*	0	0	0	1 (0.2%)	0	0

* Mutations in italic correspond to non-surveillance drug resistance mutation positions (include polymorphisms).

### Molecular transmission cluster analysis

To obtain further insight into DRM transmission, we examined transmission clusters within each cohort. Phylogenetic analysis of 1071 sequences identified 93 transmission clusters. The phylogenetic tree is shown in S3 Fig. These clusters contained 226 sequences, which accounted for 21.10% of all studied sequences. The proportion of sequences within clusters varied significantly from country to country. The minimum proportion of sequences within clusters was found in Uzbekistan (12/178, 7%) and Tajikistan (5/54, 9%), and the maximum was found in Belarus (41/158, 26%). This is most likely because the sampling densities for Uzbekistan and Tajikistan were significantly lower than those for other countries. Small clusters containing two or three sequences predominated (84 clusters out of 93). Eight mixed clusters were found, in which sequences from different countries were grouped. Mixed clusters usually included sequences from Russia and another country: Uzbekistan (3 clusters), Armenia (1 cluster), Azerbaijan (1 cluster), Belarus (1 cluster), and Tajikistan (1 cluster). One cluster was formed by sequences from Armenia and Tajikistan. Detailed information on the number and size of clusters is presented in Table 5.

**Table 5:** Cluster characteristics by number, size and country.

Number of sequences in clusters	Country						Mixed	Total
	Armenia	Azerbaijan	Belarus	Russia	Tajikistan	Uzbekistan		
2	9	6	13	31	1	6	7	73
3	1	1	3	4	1	-	1	11
4	1	2	-	-	-	-	-	3
5	-	-	-	2	-	-	-	2
6	-	-	1	2	-	-	-	3
7	-	-	-	1	-	-	-	1
Total number of clusters, n	11	9	17	40	2	6	8	93
Total number of sequences in clusters, n (%)	25 (21)	23 (24)	41 (26)	103 (22)	5 (9)	12 (7)	17	226 (21)
Total number of sequences from country, n	120	96	158	465	54	178	-	1071

Sequences with DRMs were found slightly more often in clusters (21% vs 18%, p = 0.335). In this case, we did not consider the A62V polymorphic mutation. Detailed information on the distribution of sequences with DR mutations within clusters and outside clusters for each country is presented in Table 6.

**Table 6:** Clustering of sequences with DRMs by country.

	Armenia	Azerbaijan	Belarus	Russia	Tajikistan	Uzbekistan	Total
Number of sequences in clusters, n (%)	7	3	11	23	2	1	47
	(28)	(13)	(27)	(22)	(40)	(8)	(21)
Number of sequences outside of clusters, n (%)	18	16	29	57	10	22	152
	(19)	(22)	(25)	(16)	(20)	(13)	(18)
Total number of sequences, n (%)	25	19	40	80	12	23	199
	(21)	(20)	(25)	(17)	(22)	(13)	(19)

Most often, the sequences in clusters contained the same mutations that were found among nonclustering sequences. Mutations with a frequency higher than 1% in the study cohort, in clusters and outside clusters were E138A (5.3% vs 5.7%, overall, 5.6%, p = 0.830), K103N (3.5% vs 2.7%, overall 2.9%, p = 0.515), and V106I (3.5% vs 1.5%, overall 2.0%, p = 0.054), respectively. It should be noted that K103S, with a 0.6% prevalence, was found mainly in clustered sequences (2.6% vs 0.1%, p<0.001). S3 Table provides information on all mutations identified in the studied cohort.

There were no significant differences in sex, transmission route or age between patients with sequences within clusters and patients with sequences that were not clustered.

It was also found that the time since HIV diagnosis in clustered patients was significantly shorter than that in nonclustered patients (1.26 years (159 patients) vs 2.74 years (644 patients), p<0.0001).

A significant difference was found in the proportion of clustered patients based on treatment experience. In the study, there were only 34 such patients (3.2% of the study population). Of these, a sequence from only 1 patient (0.4%) was found within the clusters. The sequence of the remaining 33 patients (3.9%) were not clustered.

## Discussion

The HIV epidemic in the EECA region is growing at a concerning rate, and conditions supporting continuous growth exist [1]; it is a high number of PLWH, poor ART adherence, stock-outs of ART drugs, widespread NNRTI-based first-line ART regimens, and weak routine viral load monitoring systems. These may be preconditions for both epidemic development and the emergence of DR.

Since EECA countries have adopted a “treat-all” and “treat-early” policy regardless of CD4 cell count and stage of infection, ART coverage has steadily increased over the past decade. Increased ART coverage without a concomitant strengthening of the control system, including DR surveillance studies, will inevitably lead to the emergence and transmission of DR viral variants [15,16,36]. Moreover, because DR testing for clinical purposes is not common in the region, PLWH with DR may not be identified and may not receive the ART regimen that would be most effective for them, which could lead to further transmission and spread of HIV DR. Therefore, it is currently extremely important to conduct surveillance studies of DR in this region.

In this study, we assessed DR in 1071 HIV-infected patients in AIDS clinics, which are major locations for ART delivery, in six EECA countries, i.e., Armenia, Azerbaijan, Belarus, Russia, Tajikistan, and Uzbekistan, at the time of treatment initiation (or reinitiation) between 2017 and 2019. According to UNAIDS estimations, more than 78% of PLWH in the EECA region live in these 6 countries [1].

Despite the fact that EECA countries are closely positioned to one another and the development of the HIV epidemic has proceeded similarly in these countries, analysis of HIV genetic variants revealed a number of distinctions, and knowledge about the current situation can be important for the creation of national policy regarding HIV. Although sub-subtype A6 is still predominant in Armenia, Azerbaijan, Belarus, and Russia, CRF63_02A1 is the most prevalent variant in Tajikistan. The epidemic in Uzbekistan is represented by two these genetic variants, A6 (48.3%) and CRF63_02A1 (49.4%), and the latest study described an increase in the prevalence of the CRF63_02A1 variant [34]. Interestingly, the greatest genetic diversity of HIV-1 was observed in Russia and Armenia.

Collected data about HIV testing and treatment in the studied countries showed that there are gaps in HIV testing, observation and treatment of HIV-infected people in these countries. All studied countries are far from achieving the 90-90-90 (or 90% - 81% of PLWH—73% of PLWH) goal. The reported prevalence of PLWH who know their status was low, with the lowest prevalence being found in Tajikistan (61%). All studied countries should expand HIV testing by approximately 10–29% depending on country rates to achieve the first 90% target. Although the number of patients receiving ART has significantly increased in recent years, the estimation of the rate of PLWH on treatment is still low. These data support the need for expanding ART by at least 20–30% to achieve the second 90% target. As a consequence of insufficient ART coverage, the number of PLWH who are virally suppressed is also far from the target of 73% of PLWH. This indicator ranged from 37% and 38% in Tajikistan and Russia, respectively, to 55% in Armenia. Thus, it is necessary to increase the number of patients receiving ART as well as to increase the effectiveness of ART.

The prevalence of PDR to any drugs in ART-initiating patients ranged from 2.8% to 16.7%, with the lowest prevalence being observed in Uzbekistan and the highest in Tajikistan. As expected, given the less common usage of first-line ART regimens and high genetic barrier, PDR to PIs was not detected in all countries. The prevalence of PDR to NRTIs was relatively low and ranged from 0% in Azerbaijan to 5.6% in Tajikistan. This fact suggests that the WHO recommendations for PrEP regimens will be effective and can be used to prevent new HIV infections in these countries [37]. More importantly, PDR to NNRTIs represented the major contribution of HIV DR and reached worrying levels of 11.1% and 11.4% in Tajikistan and Belarus, respectively, thus exceeding the 10% threshold recommended by the WHO for immediate public health action [16,17]. PDR to single drugs was the highest for NVP in all countries. Additionally, it should be noted that in most cases, there was a high level of DR. As expected, the overall PDR prevalence was higher in ART-initiating patients with prior ART drug exposure than in treatment-naïve patients.

The trends of HIV DR over time differed from country to country. The overall prevalence of PDR in Uzbekistan in this study was 2.8% for samples collected from 2017–2019, which were in accordance with a previous study that found a prevalence of 2.96% for samples collected from 2015–2016 [21]. It is important to note that the prevalence of PDR in Armenia has markedly increased during the last decade (2009–2010) from 1.5% [22] to 9.2%. In the current study, the overall prevalence of PDR in Russia was 4.5%, whereas a prevalence of 5.3% was previously described for patients with a date of diagnosis between 1998–2017 [20]; it should be noted that the mentioned Russian study assessed the prevalence of surveillance DRMs and not PDR. To our knowledge, the current study is the first to report on the PDR prevalence in Azerbaijan (4.2%), Belarus (13.9%), and Tajikistan (16.7%).

The most frequent PI DRMs included M46I and L90M, which are associated with DR to nelfinavir, which was not considered for PDR estimation and has not been used since 2013; additionally, N88D and L10F only minimally reduce susceptibility to drugs. Common NRTI DRMs included polymorphic mutations for sub-subtype A6, including A62V [20,38], M41L and L210W, which are associated with low levels of DR to AZT, and T69D, which has a minimal effect on susceptibility to drugs.

The predominant NNRTI DRMs included E138A/G, which is the natural polymorphic mutation for sub-subtype A6, was previously shown to have a prevalence of 7% frequency in Russian HIV-infected patients [20], and is associated with low-level DR to rilpivirine, which is not taken into account in the assessment of PDR; V106I, which alone causes a minimal reduction in NNRTI susceptibility; and K103N/S, which causes a large reduction in NVP and EFV susceptibility.

According to the described PDR level in the studied countries, the highest prognostic potency of ART regimens was observed in Russia, where the prevalence of DR was 0.2% for all drugs used for first-line therapy (excluding DTG, since data on this drug were not obtained in this study). Based on the obtained PDR level, it can be concluded that the currently recommended first-line ART regimens will be effective in Armenia, Azerbaijan and Uzbekistan; the highest DR level among all drugs used for first-line ART was found for EFV, but the DR level did not exceed 5% in these countries. It can be inferred that Tajikistan and Belarus should consider switching to the NNRTI-free ART regimen due to the threat of ART ineffectiveness. However, it should be noted that the WHO recently recommended the phasing out the use of EFV and NVP in first-line regimens at any PDR prevalence, and not only at the 10% threshold [39].

The proportion of clustered sequences (21.1%) was small, and the majority of the sequences were grouped into small clusters (2–3 sequences), which indicates a too low sampling density. Unfortunately, at the moment, a very small number of sequences from EECA countries have been deposited in databases, which hinders the conduct of serious molecular epidemiological studies of HIV. The introduction of the drug resistance analysis into routine medical care, which was also carried out in the framework of this study, may initiate the accumulation of molecular data on HIV variants circulating in the region.

Notably, K103N/S, which was previously described as DRM with transmission fitness similar to wild-type virus with the ability to persist for years in the infected host [40–42], was found mainly in clustered sequences (6.2%) then outside clusters (2.8%). This suggests that countries are currently experiencing an increase in the rate of spread of drug resistance to nevirapine and efavirenz.

Other DRMs were described inside and outside of transmission clusters with no reliable regularity. It was also found that the time since HIV diagnosis in clustered patients was significantly shorter than that in unclustered patients (1.26 years vs 2.74 years).

Our study has limitations that need to be acknowledged. The study did not comply with the WHO guidelines for the PDR HIV DR surveillance study because of inconsistency of the amount of ART delivery clinics involved in patient’s enrollment, sampling method, enrollment period, and total number of collected samples [17]. Additionally, the study included predominantly treatment-naïve patients. Because of this, it can be expected that the reported PDR level is probably underestimated.

However, the collected data are valuable because this is the first step to determining the main trends of DR in the studied countries. To the best of our knowledge, this is the largest study of DR in the EECA region and involved longitudinal collection of data from most parts of the countries.

The final recommendation is to implement DR surveillance studies at the national level among different cohorts of patients according to the WHO guidelines. Knowledge of PDR prevalence and patterns at the national level can be used to adapt country-wide treatment guidelines and will help improve the effectiveness of ART and reduce the prevalence of HIV DR in the long term.

## Supporting information

S1 Fig Distribution of patients by date of first positive immune blot. (TIF)

S2 Fig Prevalence of NNRTI DR mutations. (TIF)

S3 Fig Maximum likelihood phylogenetic tree of HIV-1 pol sequences. (TIF)

S1 Table Epidemiological and clinical characteristics of the study population. (TIF)

S2 Table PDR to antiretroviral drug of first-line ART preferred regimen. (TIF)

S3 Table Clustering of sequences with DRMs. (TIF)
