# Correction: Prevalence of HIV-1 drug resistance in Eastern European and Central Asian countries

**DOI:** 10.1371/journal.pone.0340124

**Published:** 2026-01-02

**Authors:** Alina Kirichenko, Dmitry Kireev, Alexey Lopatukhin, Anastasia Murzakova, Ilya Lapovok, Daria Saleeva, Natalya Ladnaya, Agigat Gadirova, Sabina Ibrahimova, Aygun Safarova, Trdat Grigoryan, Arshak Petrosyan, Tatevik Sarhatyan, Elena Gasich, Anastasia Bunas, Iryna Glinskaya, Pavel Yurovsky, Rustam Nurov, Alijon Soliev, Laylo Ismatova, Erkin Musabaev, Evgeniya Kazakova, Visola Rakhimova, Vadim Pokrovsky

S1 Table was uploaded incorrectly. Please see the correct S1 Table here.

## Supporting information

S1 Table Epidemiological and clinical characteristics of the study population. (PDF)
