# Supplementary figures and images for: Prevalence of HIV-1 drug resistance in Eastern European and Central Asian countries
Source: PLoS One. 2022 Jan 21;17(1):e0257731. doi: 10.1371/journal.pone.0257731 (PMC8782385; doi:10.1371/journal.pone.0257731)

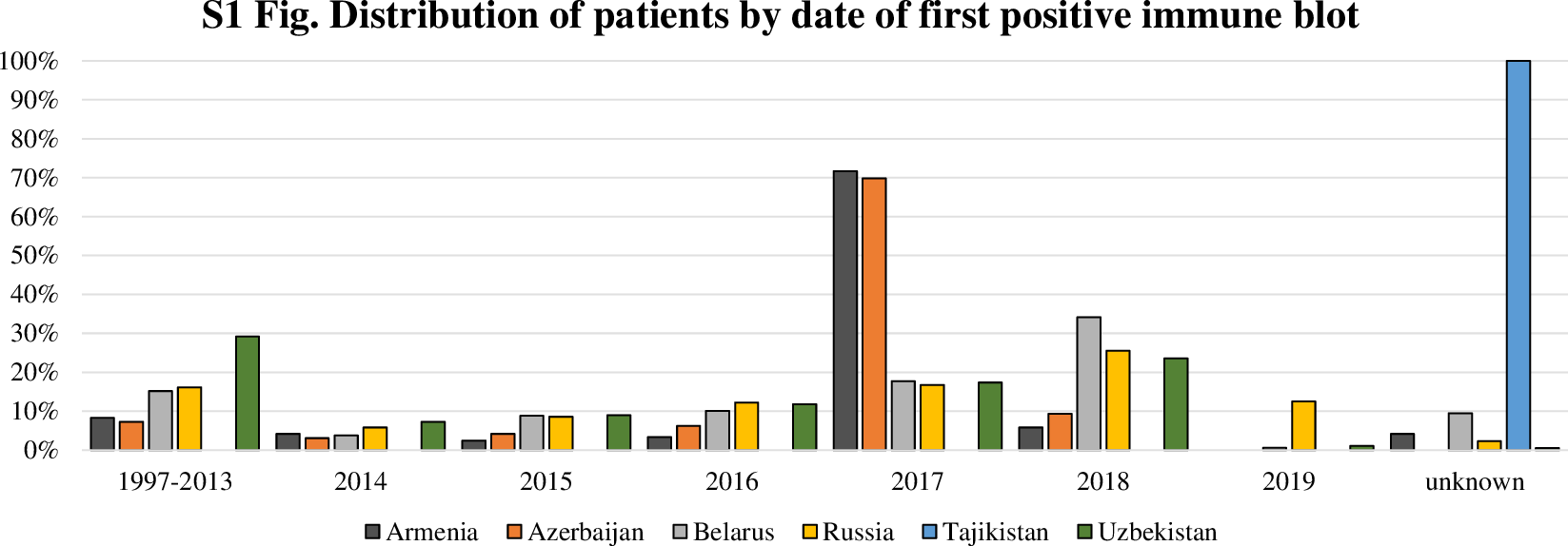

Supplement: S1 Fig — (TIF) [file pone.0257731.s001.tif]

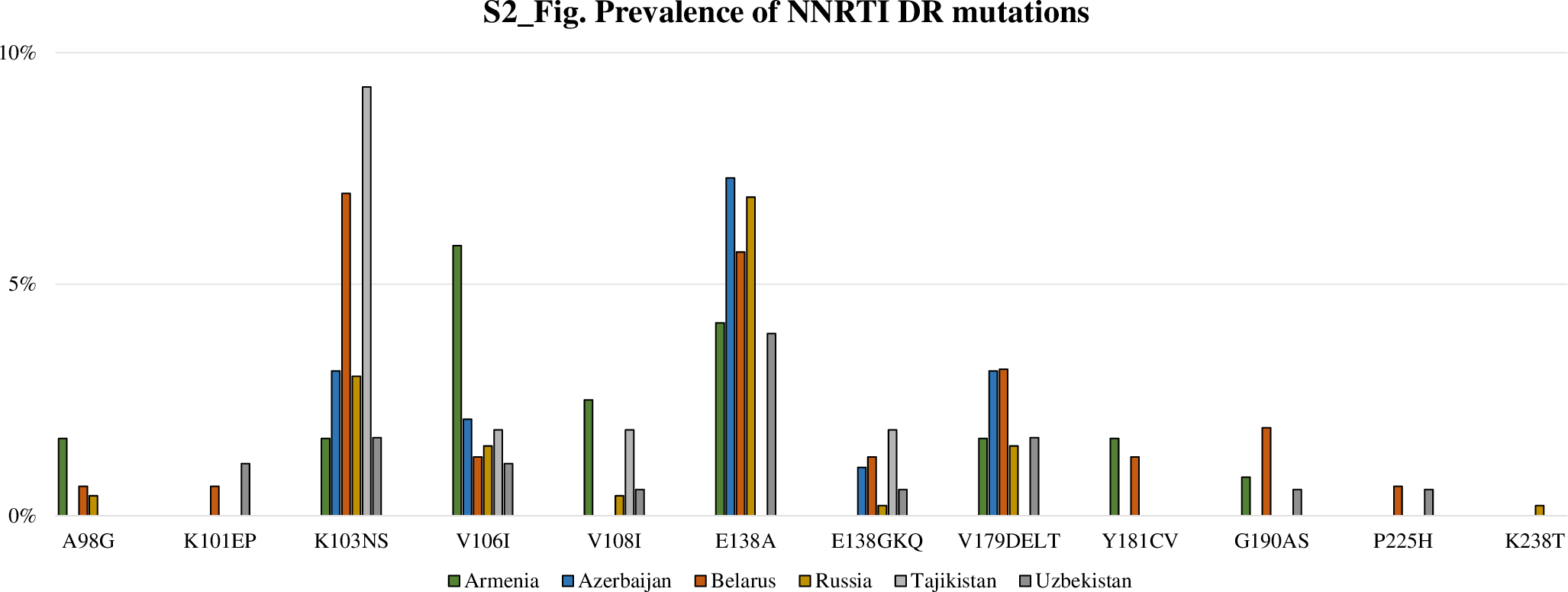

Supplement: S2 Fig — (TIF) [file pone.0257731.s002.tif]

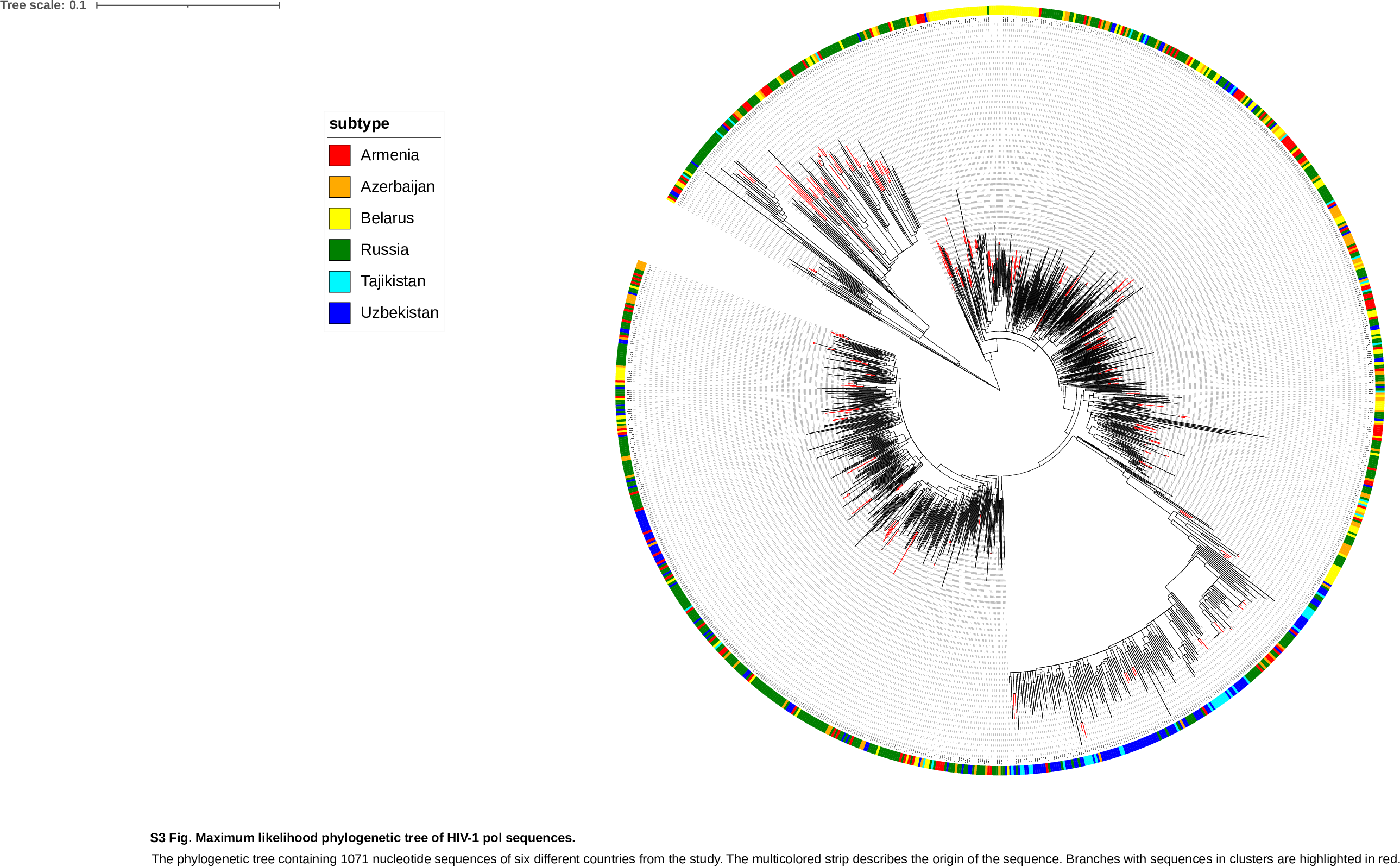

Supplement: S3 Fig — (TIF) [file pone.0257731.s003.tif]

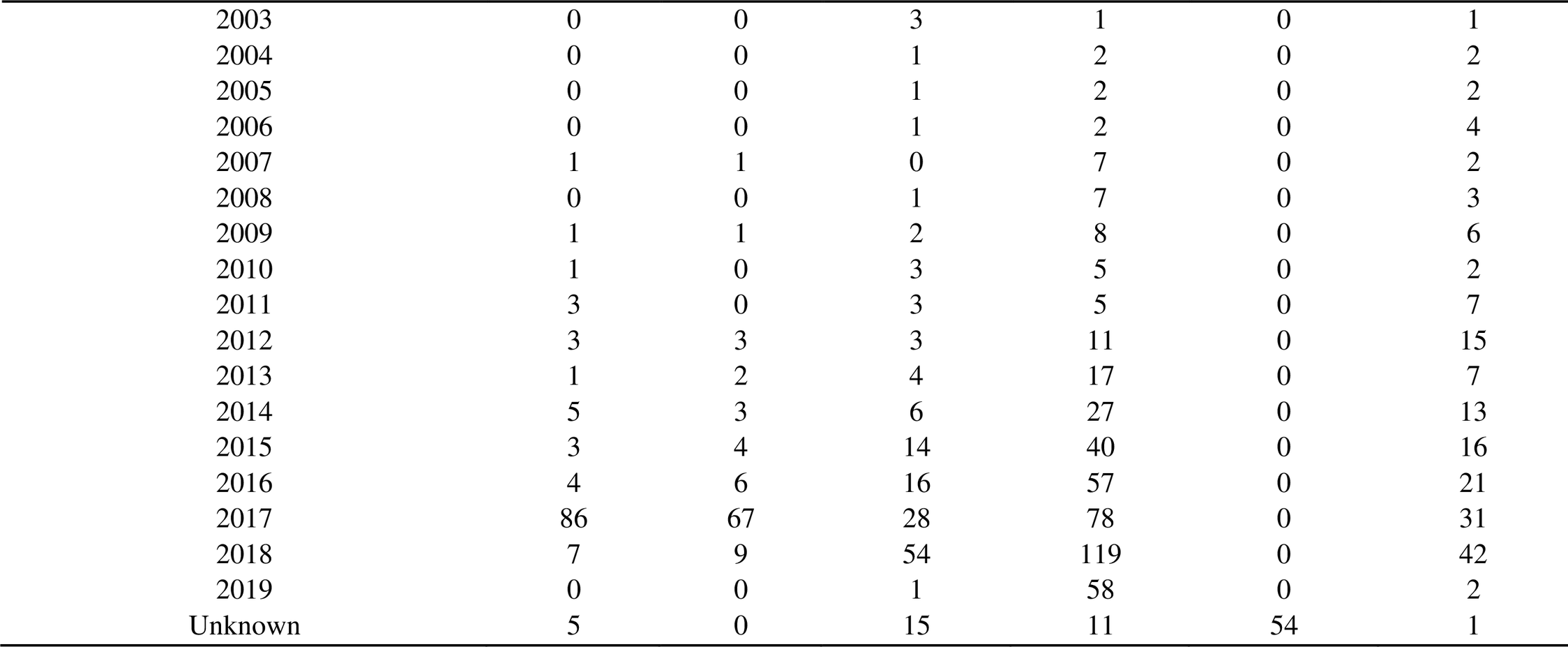

Supplement: S1 Table — (TIF) [file pone.0257731.s004.tif]

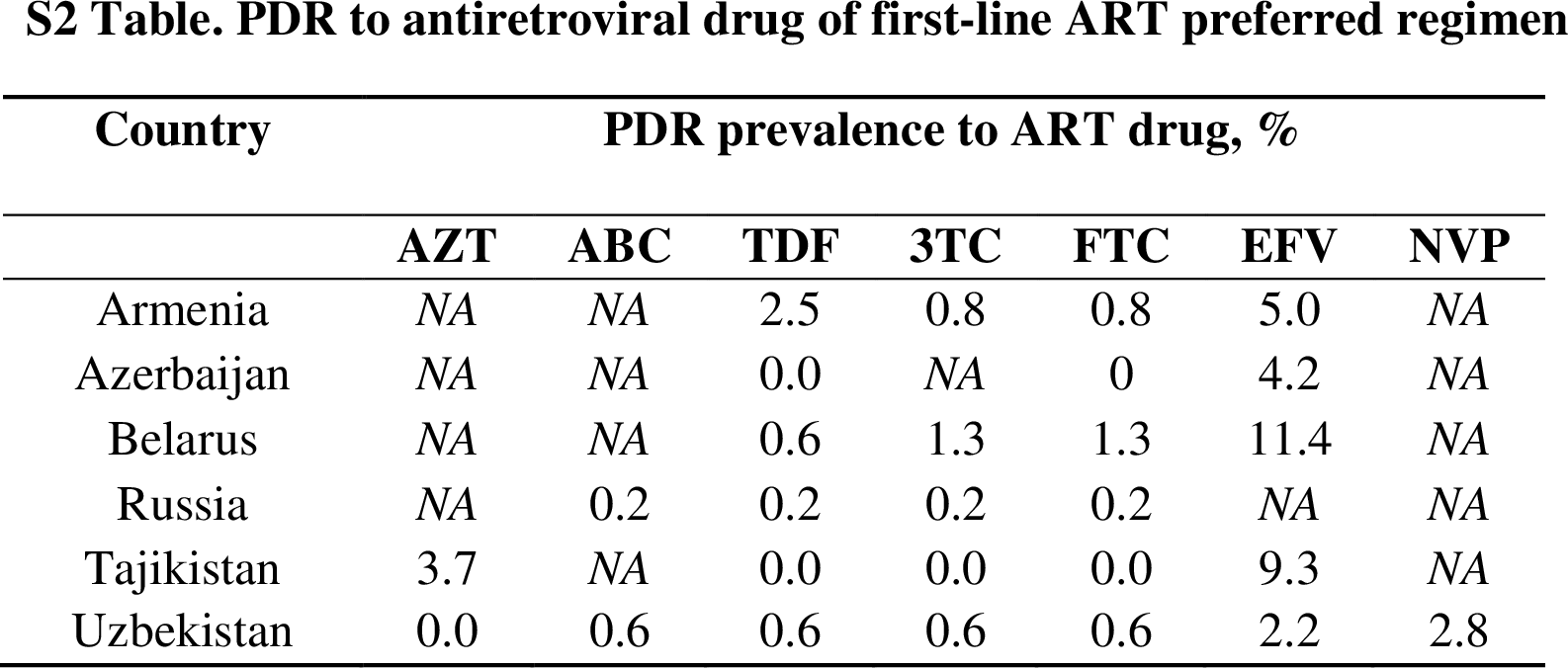

Supplement: S2 Table — (TIF) [file pone.0257731.s005.tif]

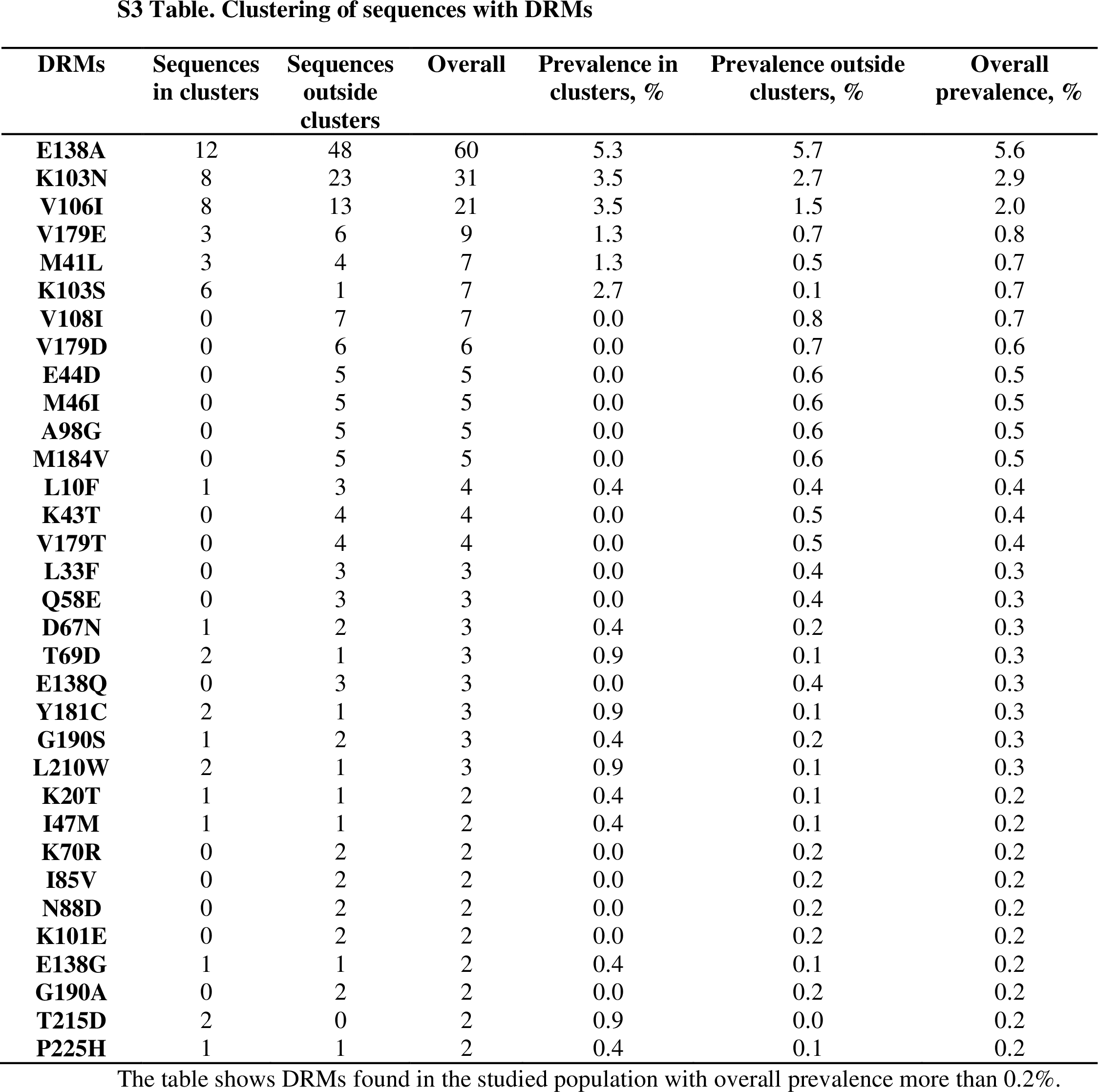

Supplement: S3 Table — (TIF) [file pone.0257731.s006.tif]
