# Supplementary material for: Correction: Prevalence of HIV-1 drug resistance in Eastern European and Central Asian countries
Source: PLoS One. 2026 Jan 2;21(1):e0340124. doi: 10.1371/journal.pone.0340124 (PMC12758738; doi:10.1371/journal.pone.0340124)
Supplement: S1 Table — (PDF) [file pone.0340124.s001.pdf]

S1 Table. Epidemiological and clinical characteristics of the study population

Characteristic	Country of origin					
	Armenia	Azerbaijan	Belarus	Russia	Tajikistan	Uzbekistan
**Number of patients**	120	96	158	465	54	178
**Median age, years (IQR)**	37 (29-45)	38 (30-44)	35 (31-40.5)	34 (29-39.8)	33.5 (28-39)	37 (31-44)
**Sex, n (%)**						
Male	79 (65.8)	62 (64.6)	117 (74.0)	315 (67.7)	39 (72.2)	89 (50.0)
Female	41 (34.2)	34 (35.4)	39 (24.7)	147 (31.6)	15 (27.8)	89 (50.0)
Unknown	0	0	2 (1.3)	3 (0.7)	0	0
**Route of HIV transmission, n (%)**						
Sexual (overall)	108 (90.0)	80 (83.4)	81 (51.3)	261 (56.1)	32 (59.3)	132 (74.2)
Heterosexual	102 (85.0)	76 (79.2)	24 (15.2)	137 (29.5)	32 (59.3)	132 (74.2)
MSM	6 (5.0)	4 (4.2)	0	33 (7.1)	0	0
Sexual (unspecified)	0	0	57 (36.1)	91 (19.5)	0	0
IDU	11 (9.2)	14 (14.6)	47 (29.7)	72 (15.5)	7 (13.0)	16 (9.0)
Mother-to-child	0	1 (1.0)	1 (0.6)	3 (0.6)	2 (3.7)	0
Outbreak	0	0		5 (1.1)	0	12 (6.7)
Unknown	1 (0.8)	1 (1.0)	29 (18.4)	124 (26.7)	13 (24.0)	18 (10.1)
**Viral load (log10 copies/mL), median (IQR)**	5.2 (4.3-5.9)	-	4.7 (4.1-5.1)	4.6 (4-5.1)	5.1 (4-5.9)	4.4 (3.8-4.9)
**CD4+ T-cell count (cells/mm3), median (IQR)**	325 (78-504)	-	452 (264-677)	440 (287-587)	252 (122-358)	273 (150-454)
**ARV experience, n (%)**						
Yes	7 (5.8)	0	6 (3.8)	0	7 (13.0)	14 (7.9)
No	113 (94.2)	96 (100.0)	152 (96.2)	465 (100.0)	47 (87.0)	164 (92.1)
**Date of first positive immune blot, n**						
1997	0	0	0	1	0	0
2000	0	0	0	1	0	0
2001	0	0	1	4	0	0
2002	0	0	1	2	0	1

2003	0	0	3	1	0	1
2004	0	0	1	2	0	2
2005	0	0	1	2	0	2
2006	0	0	1	2	0	4
2007	1	1	0	7	0	2
2008	0	0	1	7	0	3
2009	1	1	2	8	0	6
2010	1	0	3	5	0	2
2011	3	0	3	5	0	7
2012	3	3	3	11	0	15
2013	1	2	4	17	0	7
2014	5	3	6	27	0	13
2015	3	4	14	40	0	16
2016	4	6	16	57	0	21
2017	86	67	28	78	0	31
2018	7	9	54	119	0	42
2019	0	0	1	58	0	2
Unknown	5	0	15	11	54	1
